# How does decentralisation affect health sector planning and financial management? a case study of early effects of devolution in Kilifi County, Kenya

**DOI:** 10.1186/s12939-017-0649-0

**Published:** 2017-09-15

**Authors:** Benjamin Tsofa, Sassy Molyneux, Lucy Gilson, Catherine Goodman

**Affiliations:** 1 KEMRI Wellcome Trust Research Programme, KEMRI Centre for Geographic Medicine Research Coast, P.O. Box 230-80108, Kilifi, Kenya; 2 Global Health Department, Faculty of Public Health and Policy London School of Hygiene and Tropical Medicine, London, UK; 3 Health Economics Unit, School of Public Health and Family Medicine, University of Cape Town, Cape Town, South Africa; 4 Centre for Tropical Medicine and Global Health, Nuffield department of Medicine, University of Oxford, Oxford, UK

**Keywords:** Decentralization, Devolution, Planning and budgeting, Financial management, Kenya

## Abstract

**Background:**

A common challenge for health sector planning and budgeting has been the misalignment between policies, technical planning and budgetary allocation; and inadequate community involvement in priority setting. Health system decentralisation has often been promoted to address health sector planning and budgeting challenges through promoting community participation, accountability, and technical efficiency in resource management. In 2010, Kenya passed a new constitution that introduced 47 semi-autonomous devolved county governments, and a substantial transfer of responsibility for healthcare from the central government to these counties.

**Methods:**

This study analysed the effects of this major political decentralization on health sector planning, budgeting and overall financial management at county level. We used a qualitative, case study design focusing on Kilifi County, and were guided by a conceptual framework which drew on decentralisation and policy analysis theories. Qualitative data were collected through document reviews, key informant interviews, and participant and non-participant observations conducted over an eighteen months’ period.

**Results:**

We found that the implementation of devolution created an opportunity for local level prioritisation and community involvement in health sector planning and budgeting hence increasing opportunities for equity in local level resource allocation. However, this opportunity was not harnessed due to accelerated transfer of functions to counties before county level capacity had been established to undertake the decentralised functions. We also observed some indication of re-centralisation of financial management from health facility to county level.

**Conclusion:**

We conclude by arguing that, to enhance the benefits of decentralised health systems, resource allocation, priority setting and financial management functions between central and decentralised units are guided by considerations around decision space, organisational structure and capacity, and accountability. In acknowledging the political nature of decentralisation polices, we recommend that health sector policy actors develop a broad understanding of the countries’ political context when designing and implementing technical strategies for health sector decentralisation.

**Electronic supplementary material:**

The online version of this article (doi:10.1186/s12939-017-0649-0) contains supplementary material, which is available to authorized users.

## Background

Health sector planning, budgeting and efficient financial management are key to ensuring rational prioritization and use of limited resources, and in responding to community priorities, broader political interests, and the fiduciary requirements of national bodies and external funders [1, 2]. However, a major and constant challenge has been the misalignment between identified sectoral policies, technical planning and budgetary allocation; and at the same time ensuring full community involvement and participation in the priority setting activities [3–5]. The misalignment between planning and budgeting within the health sector in many developing countries has often been as a result of institutionalised separation between these processes [5]. This problem has resulted in an inability of the health sector to influence additional resource allocation in the broader government resource allocation processes; and could explain why most developing countries are constantly unable to achieve their health sector medium term goals [4, 6].

To address these dual challenges of misalignment between planning and budgeting, and poor community involvement, health system decentralisation has for many decades been promoted as a priority reform agenda [7, 8]. Decentralisation involves the transfer of decision making power and authority over management of public affairs from a central level of government to sub-national levels. It has been argued to promote community participation, accountability, and technical efficiency in the management of public resources [8, 9]. The transfer of power and authority may involve revenue generation, priority setting, resource management and/or decision making, and the sub-national units may be elected directly by the population, or appointed by the central level or by private entities [7, 10]. These multiple modes of decentralisation make it a very complex concept to study in a real world setting [8].

The call for health system decentralisation dates back to the days of the *Alma Ata declaration* in 1978, which advocated for participation and involvement of communities in managing their health affairs. At this time, the potential for decentralisation to enable community empowerment, a concern for marginalised and disadvantaged communities to have greater influence over their health services, was in essence an equity issue [11]. However, decentralisation has also been critiqued for its potential to allow increasing inequities in service provision between more and less wealthy areas and populations [12]. The publication of the World Development Report 1993: *Investing in Health*, in the early 90s [11, 13] also provided renewed momentum for decentralisation, as a ‘good governance’ intervention for improving technical efficiency in health sector resource allocation and management, including through improving community participation to allow resource management decisions to be made closer to the targeted communities [14, 15].

Empirical findings on the effects of decentralisation on health sector planning, budgeting and financial management have been varied. Decentralization has been linked with enhanced local level internal health sector resource mobilization through allowing districts to make local decisions on user fees [16]. The use of both discretional block grants, and conditional grants as mechanisms for resource allocation to decentralised units has been reported in many countries [17–21]. Increase in discretional authority over local level health sector priority setting has been linked with reduced allocations for Primary Health Care (PHC) in decentralized units in some counties [17]. It is therefore evident that, despite its growing popularity as an approach to tackling poor health system governance, the experiences of health sector decentralisation in most developing countries have been varied, irrespective of the form or mode of decentralisation adopted [7, 8]. There is also wider recognition that, by aiming to transfer *power* from one set of actors to another, decentralisation is an inherently highly political process whose effects and outcomes are heavily influenced by contextual factors [14, 22].

The decentralization debate has dominated the political arena in Kenya since independence. This has seen the country adopt several decentralisation policies and strategies over time [23, 24]. Within the Kenyan health sector, user fees were introduced in public health facilities in 1989, and District Health Management Teams (DHMTs) and District Health Management Boards (DHMBs) were established to oversee management of these fees [5, 25, 26]. A bottom-up health sector Annual Planning process was introduced at the same time. In 2009 the MoH introduced the Health Sector Services Fund (HSSF) which is a system where the government finances some recurrent costs for primary level health facilities by sending monies directly from National Treasury to health facility bank accounts, without going through the traditional disbursement bureaucracy in the health system [27]. Most recently, in August 2010, Kenya adopted a new constitution that created a devolved government system with 47 semi-autonomous counties to be established after the 2013 general elections. This devolution was largely driven by larger countrywide political processes with a broader goal of enhancing equitable resource allocation amongst regions and communities, and public community involvement in public resource management. The health service delivery function was among the key services earmarked to be devolved [28].

The 2010 constitution established four mechanisms through which counties are resourced: (i.) the Equitable unconditional share from national government set at a minimum of 15% of all national government revenue; (ii.) an Equalisation fund allocated to marginalised counties to provide specific social services, set at a minimum of 0.5% of national government revenue; (iii.) Local revenue generated within the county through county level taxes; and (iv.) Conditional grants given by national government to counties to address specific national strategic priority issues. A Public Finance Management (PFM) Act elaborates the overall government planning and budgeting, and financial management process including key events and specific time lines at national and county level (Table S1).

In this paper, we examine the early effects of devolution in Kenya on health sector planning, budgeting and financial management. Specifically, we examine the extent to which devolution addressed the historical challenges of a mismatch between health sector planning and budgeting, and inadequate community involvement. With the growing acknowledgment that decentralisation effects and outcomes are highly context specific, this paper provides empirical findings from the Kenyan context. This paper is also unique in reporting on data collected during the early days of decentralisation implementation rather than in a more stable phase, providing a deeper understanding of health systems functioning during the process of major political change.

## Study methods

This is a qualitative case study focusing on Kilifi County, but also drawing on data from the broader implementation of devolution in across the country. Kilifi county has a population of approximately 1.2 million people and covers an area of 12,246 km^2^. About 74% of the population live on less than one dollar a day [29]. The decision to use one county was to allow for a deeper exploration of the issues under focus within the study. To help in interpreting the findings from Kilifi, we also incorporated national level data collection through national level key informant interviews, and we also drew from national level and country-wide non-participant observation of the unfolding events with the implementation of devolution across the country.

Kilifi County, is one of the six counties that formed the former Coast Province. The Coastal region of Kenya is largely believed to have been the originator of the decentralization debate in Kenya, which championed for a federal government system at independence [23, 30]. Kilifi County is also part of the broader health systems governance learning site, within which this study was nested [31].

### Conceptual framework

**Fig. 1:**
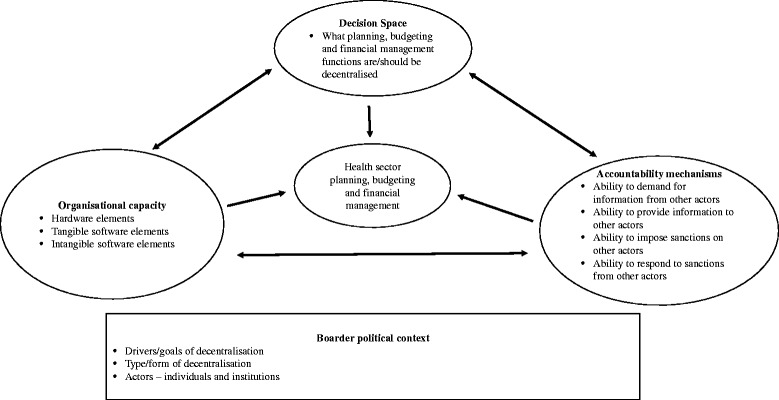
Study conceptual framework

We adapted a conceptual framework proposed by Bossert and Mitchel (2011) which suggests that *decision space*, *accountability* and *organizational capacity* often interact to affect and influence the range of choices made by decentralised units within decentralised settings [32]. In our adaptation, we also considered the broader political context including the mode of decentralisation, its overall goals and the key actors and players driving its design and implementation (Fig. 1). For decision space we considered it as the degree of discretion that peripheral units have within the law, and their ability to ‘bend the law’ [8]. We considered accountability as the ability of actors to demand from or provide information to others within the system, and to impose or respond to sanctions [33]; and we considered organisational capacity around the dimensions of *hardware (*infrastructure, technology and finances), *tangible software (*organisational systems, management systems, processes and procedures), and *intangible software* (communication, relationships, norms and values, and power), all of which are necessary for optimal functioning of health system organisations [34, 35].

We used this framework to inform the development of our data collection tools and to frame our interpretation of our study findings and the discussion.

### Data collection

We used several qualitative data collection techniques, including participant and non-participant observation, regular reflective practice, document reviews and key informant interviews. Data were collected between December 2012 to December 2014, just before and during the early days of implementation of the devolved government systems.

Data collection was facilitated by BT who has a long history of working with health system managers both in Kilifi County and at national level. He worked for 3 years as the district manager of health in Kilifi prior to devolution, and during 2012 was based fulltime at the national Ministry of Health (MoH) as a technical adviser to the Technical Planning Department. During the data collection period for this study, he was co-opted into the national health sector technical committee that was charged with designing and facilitating the transfer of functions from the national MoH to the County Departments of Health (CDoH). In Kilifi he was formerly invited by the county managers to support the planning process in the county.

BT’s previous experience in the health sector, and his engagement with the MoH at county and national level during the data collection phase provided him with a unique insider perspective, with access to information and operations of the health system both at national and Kilifi County level that would not be accessible to purely external researcher [36]. To strengthen objectivity in the interpretation of his observations, regular formal reflective sessions were carried out with the other research team members to allow for group reflections on the findings. Field notes were kept in the form of a diary throughout this period. The observation field notes, and deliberations from the reflection sessions were triangulated with data from the key informant interviews and documents reviews so as to minimize any possible bias from the insider status

#### Observations and reflective practice

County level observations were carried out as part of a longer term programme of health system governance research - a ‘learning site’ - established in Kilifi in 2012 [37]. A health system governance learning site is a geographic setting where health systems managers and researchers work together over a relatively long period to identify operational governance challenges and questions, and to seek solutions and answers to the identified challenges. Two other learning sites, linked with the Kilifi one, have been established in Cape Town and Johannesburg in South Africa [35, 37]. As part of the research activities within the learning site, regular formal reflective practice sessions are conducted among the researchers, between the researchers and health managers, and across learning sites. Both BT and SM are part of the Kilifi learning site, while LG is part of the Cape Town learning site. National level and country-wide observations were done by BT through participating in meetings and activities aimed at facilitating the transfer of functions from national level MoH to the counties, and through noting and diarizing key events in the implementation of decentralisation and their subsequent health sector effects across the country.

#### Document reviews

The content of all documents relating to the design and implementation of the devolved government system generally, and health sector, planning and budgeting more specifically, was reviewed to provide an understanding of the overall goals of the devolved government system, and processes for health sector planning and budgeting under devolution. These documents included the 2010 constitution, the County Government Act 2012, the Public Finance Management Act 2012, the Draft Kenya Health Policy (KHP) 2012–2013, the Kenya Health Sector Strategic Plan (KHSSP) 2013–2017, the Health Sector Planning and Budgeting Tools and Guidelines, and the Draft Kilifi County Integrated Development Plan (CIDP) 2013–2017.

#### Semi-structured interviews

We conducted 28 semi-structured interviews in English with a wide range of purposively selected participants with a key role in the implementation of devolution at national level and in Kilifi County. We included those involved in general as well as health sector planning and budgeting specifically. These interviews were largely directed by the data collected through the methods highlighted above, and were complemented by numerous informal discussions with key participants throughout the study period, as part of broader learning site interactions. At national level interview participants were drawn from the national MoH, non-government health development partners, UN agencies, the Commission for the Implementation of the Constitution, the Transition Authority, and the national assembly committee on health. At the county level, participants were drawn from the CDoH, County Treasury, County Public Services Board, County Assembly, and the County Transition Authority coordination office. All semi-structured interviews were tape recorded and later transcribed verbatim.

### Data management and analysis

Interview transcripts, observations and reflective practice notes were imported into N-Vivo 9 software for charting and coding. They were later analysed using the thematic framework approach [38], identifying main themes guided by our study conceptual framework, and those emerging from the data.

## Study results

In our results, we begin by providing an overview of the overall country-wide county government structure, and the planned and actual process of transferring devolved functions from national to county level. We then elaborate on how the health sector planning and budgeting process should in theory work in general across the country, and what happened in practice in the 2013/14 fiscal year, with more focus on Kilifi County, and the key country-wide, and county specific influences on this process that year. We conclude our findings by highlighting some of the early outcomes of devolution on health sector financial management processes in Kenya.

### County government structure and transfer of county functions

The constitution established a county government with two arms: an executive with an elected County Governor and Deputy Governor; and a 10-member County Executive Committee (CEC) appointed by the governor. A legislature known as the County Assembly (CA) was also established, made up of elected Members of County Assembly (MCAs) representing each electoral ward in the county, and as nominated by political parties in the assembly to represent special groups. The CEC members have overall policy and political responsibility over each of the ten County Departments, including health. Within each department and working under the CEC member is a Chief Officer, also appointed directly by the governor, with overall accounting and administrative responsibility over the respective department [39].

The constitution outlined a seven year process of transferring functions from national to county governments, running from August 2010 [28, 40]. However, reacting to growing pressure from the county governments, the president in June 2013 directed that all government functions to be undertaken in counties be transferred immediately. This happened at a time when most county governments had not fully established their structures to undertake these functions.

### County government planning and budgeting under devolution

The national level planning and budgeting events relevant to the health sector are outlined in Additional file 1: Table S1. At the county level, once established, each county government should establish a County Treasury, which facilitates and oversees planning and budgeting, and overall management of public finances. It should establish a consolidated County Revenue Account, to hold all county revenue received from national government or raised locally. Any withdrawals or payments from this account require approval from the office of the Comptroller of Budgets at the National Treasury who is responsible for ensuring that county governments adhere to government wide financial regulations. Key county planning and budgeting events relevant to the CDoH planning process are outlined in Additional file 1: Table S2.

All county departments should develop strategic plans, which are consolidated to form the County Integrated Development Plan (CIDP). The CIDP consolidation process should incorporate grassroots public participation, and be implemented by each county department through Annual Work Plans (AWPs). Resource allocation to all county departments should occur through an annual resource bidding process, guided by the departmental AWPs. The county departments then develop departmental budgets, which are consolidated to form the overall annual county budget. The CEC member for finance should present the consolidated county budget to the full CEC for approval before submitting it to the CA by the end of April each year. The CA should invite submissions and inputs from members of the public and other stakeholders, with budget approval by end of June. Once approved the county treasury is required by law to publish and publicize the budget for general public information.

**Fig. 2:**
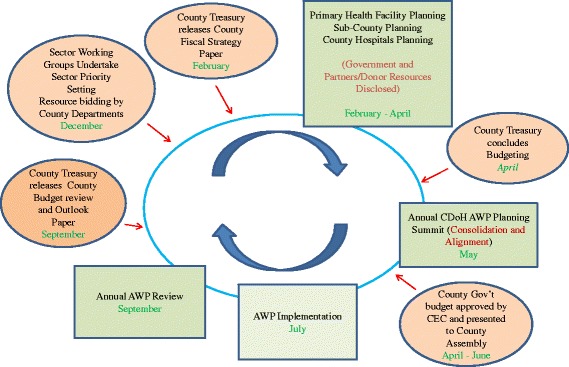
An illustration of the CDoH planning and budgeting cycle

Figure 2 shows the CDoH planning and budgeting cycle, showing the key events to be carried out within the CDoH (green boxes) and how they link with the key events in the overall county budget process coordinated by County Treasury (orange circles). The CDoH’s AWP process should ideally begin in September with a performance review of the previous year’s AWP and an elaboration of the subsequent year’s priorities. These priorities should guide the CDoH resource bidding process once the county treasury publishes the County Budget Review and Outlook Paper which gives a detailed outline of the projected county resource basket made of allocations from national government, and locally generated revenue. It also outlines indicative allocations to county departments. Within the CDoH, the AWP planning and budgeting process is overseen by the County Chief Officer for Health with the County Health Management Team (CHMT).

Once they receive the circular providing the overall guidance, the CDoH is required to develop AWP guidelines to facilitate AWP preparation within all its planning units. The AWP tools should adhere to the planning and budgeting guidelines released by treasury, and be aligned with the national health policy and strategic agenda outlined in the KHP 2012–2030 and KHSSP 2013–17.

Once all the planning units have undertaken the AWP planning, the CHMT should convene a meeting with all health stakeholders including implementing and funding partners, and the CA Health Committee, to consolidate the work plans and outline the budget. The consolidated departmental plans and budget are then submitted to the County Treasury for incorporation into the draft county government budget for submission to the full CEC, and later to the CA for approvals.

### Early experiences of county level planning, budgeting and financial management experiences under devolution; and their influences

#### Overview of the 2013/14 planning and budgeting process in Kilifi

The Kilifi CHMT began the county health strategic plan development process in early April 2013, but then abandoned it until May 2014 because of lack of a Chief Officer in the CDoH at the time to lead and guide the process. The CDoH thus did not actively participate in the overall county budgeting process. The County Treasury went ahead to develop generic budgets for all departments including that for the CDoH, in order to avoid delaying the county budgeting process which has legally entrenched timelines. There was thus no sector AWP developed within the CDoH by the time the overall county budget process for 2013/14 was concluded.

The lack of active participation of the CDoH meant that there was hardly any community or stakeholder involvement in the health sector planning and budgeting process. However once the consolidated county budget was finalised, there was an attempt by the County Treasury to subject the budget to stakeholder and public reviews before it was finally presented to and debated by the CA for approval.

#### Influences on the 2013/14 CDoH planning and budgeting process and outcomes

##### Delays and tensions in establishing CDoH structures in Kilifi county

A major influence on the planning and budgeting process in Kilifi specifically was the delays and tensions in establishing county structures. The national MoH appointed and seconded interim County Health Coordinators to every county a few weeks before the general election in early 2013 with the mandate to set up interim county health coordination structures. These county coordinators established interim CHMTs. In Kilifi, the interim CHMT designated the three former DHMTs within the county as interim Sub-County Health Management Teams (SCHMTs) and former Hospital Management Teams (HMTs) for the three referral hospitals as the interim HMTs. There were however no clear terms of reference or guidelines provided by national or county governments for the composition, roles and mandates of these structures.

For more than a year, the governor did not appoint departmental (including health) Chief Officers. This was largely due to heightened political canvasing within the county at the time. Only the Chief Officer for Treasury was appointed in May 2013, as an interim measure to assume accounting responsibilities for all county departments. For the CDoH, an earlier appointment had led to tense working relationships between senior managers which contributed to the stalling of the CDoH Strategic Plan development process and the AWP development that had begun in early 2013. Chief Officers were subsequently appointed in April 2014.

##### Lack of clarity between CDoH and national MoH roles country-wide

At the time of the 2013/14 health sector planning and budgeting process, the proposed process of transfer of functions had not been agreed upon between national and county governments. The roles the CDoH would undertake *vis a vis* the national MoH with respect to health service provision was therefore not clear. These observations were also made by some interviewees.

[…. I was lucky enough to participate in the budgeting process for the county, so and rumour had it that commodity, some people were saying that commodity procurement will still be done at the central government. It wasn’t clear by the time we were doing this year’s budget. It wasn’t clear…] KII C 004

##### Lack of capacity of key actors to undertake their planning and budgeting roles at the county level

Beyond the structural delays and lack of clarity in roles, some county level respondents felt that the individuals and structures tasked with different planning and budgeting roles at the county level lacked the basic capacity to comprehend and undertake these roles:

[…Then again the capacities of the CECs for example are moving now to developing strategic plans and developing sector plans, we have had a problem up to now. We do not have up to today sector plans. Sector Strategic plans. Even the AWPs, they’re doing work plans….. …..this is a primary school teacher who was picked from the classroom and made CEC, and she has no capacity to develop that and you’re telling them today develop a strategic plan….] KII C 002

Several interview participants at the County and Sub-County level also pointed out that the MCAs lacked the relevant capacity to undertake their oversight roles.

[…The County Assembly for me is a body which has the will and the power to do things right but has no capacity to do it, but they have the power, they have the everything, the will and everything but the capacity is very limited because if you to have to hold me accountable, you should be analytical. You should be a person who can understand things to a certain level…] KII C 002

Because of this lack of capacity of the MCAs, there was a view that in Kilifi, the county executive could easily buy their way from the CA if they need any decision to be made; thus weakening public participation and accountability in governance processes.

[…. You see. So public participation is very weak, very weak because when you bring a Bill, you bring them [MCAs] here to take them through … you actually invite the committee to take them through the Bill. Pay them a sitting allowance; When they get it there at the assembly, they will not raise a finger on it….] KII C 002

### Early experiences in CDoH financial management processes in Kilifi County

#### Re-centralization of financial management roles at county level

The lack of a Chief Officer for the CDoH for the better part of 2013 caused significant delays in accessing funds by the service units, thus hampering service delivery within Kilifi county. As an interim measure, all financial requests for routine recurrent expenses by service delivery and coordination units had to be taken to be approved by the Chief Officer Treasury, causing significant delays in financial procedures.

[….right now as Kilifi the only challenge we have is the fact we don’t have a chief officer ….,… then these people have to come all the way from Malindi, and Mariakani; the Malindi [Sub-Counties] ones are …they have to come here, you know…] KII C 002

[…We took the voucher since before Christmas, to the county treasury, around December, (this interview was in March of subsequent year)…..yes they have not been able to pay the vouchers. So petrol also the same. We have to go and kneel down there before the supplier we cry if there is no phone and imagine if you don’t have even recurrent money even to buy airtime, it means you have to go physically if you miss him you have to borrow airtime again so we are just running in debts…] KII SC 005 ﻿However even after the appointment of the Chief Officer Health in April 2014, the delegation and transfer of financial management responsibilities for recurrent activities at Sub-County, Hospitals and PHC facilities did not happen. Sub-county and health facility managers still have to travel long distances to the County headquarters to get approvals and financing for their recurrent expenses serviced by the CDoH Chief Officer.

##### User fee lock-down in hospitals within the county

Given the challenges and delays in accessing county level funds for service delivery by the hospitals, the user fees (which the hospitals continued to collect after devolution) were a potential alternative source of funds for these facilities. However, once the County Treasury was established in Kilifi, it directed that the user fees fell under what is collectively described as ‘County Revenue’ and thus the hospitals should close their respective user fees bank accounts and be banking the money in the County Consolidated Revenue Account.

The CDoH was not happy with this directive, and for a whole year the hospitals continued to collect the money and bank it in the hospitals’ accounts, but could not spend it because of lack of authority to spend from the county treasury; even with the existing acute and emergency financial needs in these hospitals at the time.

[…We are banking 100% according to the policy and… Several millions like here I think it’s more than 30 million is inside there, frozen; and yet we have the ambulance…that cannot be removed from the fundi (mechanic), imagine……] KII SC 005

The delays in accessing funds for addressing emergency needs from the county treasury and the stand-off over the use of the user fees funds, led some hospital managers to decide to spend the money they collected from user fees, without banking them, so as to address their emergency needs without seeking approvals.

[…now sometimes (XXX hospital) has been forced to spend money at source [without banking it] as I speak now they have spent almost 500,000 shillings at source. Yeah because how do you survive, we don’t have water, we don’t have electricity you have no supplies, patients don’t have food and you have debt of around 2 million just for food alone, and such kind of things we are talking about.…] KII SC 007

##### Access to HSSF funds for PHC facilities

Prior to devolution, an additional source of funds to support service delivery particularly for primary health facilities across the country was the HSSF. As noted above, these were funds put together as a contribution by national government and two main donor agencies, DANIDA and the WB that would be sent into PHC facilities directly from central level treasury to cater for recurrent expenses [27]. However, in the early days of devolution, there were contestations over the roles of national and county government in the management of these funds. County governments wanted to undertake the selection and gazettement of the Facility Management Committees comprising of local community representatives and who were key in the management of these funds at the facility level, arguing that managing PHC facilities fell within their mandate.

Another contention was regarding the channel and flow of HSSF funds. The national MoH wanted the flow to remain as prior to devolution, i.e. move directly from national treasury to facility bank accounts. This position was supported by the WB. The county governments however wanted the funds to flow through the County Treasury, a position supported by DANIDA. These contestations were also recounted by some interviewees.

[…am hearing that there is still a tug of war between the county and national government. The county wants to gazette, to do the gazettement because they claim this is a county function. The national government also wants to do that…] KII SC 02

These contestations led to a significant delay in the release of funds. Ultimately when an agreement was reached for counties to gazette the FMCs but for the funds to go straight into facility accounts, DANIDA disagreed and withheld their contribution into the HSSF kitty, leading to a significant reduction of the total amount of funds sent directly to health facilities.

## Discussion

The design and model of the devolved government structure introduced in Kenya under the 2010 constitution created opportunities for a major increase of decision space over public resource management from national level to the county level. In addition, the devolution laws created an elaborate mechanism to increase direct and in-direct (through elected representatives) community involvement in planning and budgeting. However, these opportunities did not improve the historical challenges of poor alignment of health sector planning and budgeting processes, and poor community involvement, at least during the early days of implementation of devolution in Kilifi County. This was largely due to a lack of clarity in roles between the national MoH and the CDoHs country-wide, delays in setting up the county level structures required to facilitate the processes, and lack of the required organisational and individual capacity at county level to undertake the planning and budgeting function. All of this took place in a context of country-wide rushed transfer of functions from national to county level, in turn linked to broader political contestations in the country. In addition, the early days of decentralizing witnessed perverse re-centralisation of operational financial management processes at county level, and disruption of traditional operational financial flows to hospitals and PHC facilities.

From our findings, it is evident that within a decentralised setting, discretion over planning, budgeting and financial management often interact with organisational capacity, and accountability mechanisms, to affect health sector planning and financial management outcomes. These observations are largely in line with those of Bossert and Mitchell (2011) [32]. However, in addition, our findings suggest that this interaction and associated outcomes need to be viewed within the broader political context in which the decentralisation model was designed and implemented.

From applying our adapted conceptual framework, we see that the structure and implementation process of decentralisation laws and policies in Kenya has always been influenced and informed by the country’s political history and culture [41]. Key features of this history include the political heritage of the main political parties and key political actors in the country over time, the perceived injustices and inequalities across different tribal and geographical populations, and the experience of political violence especially after the 2007 general election [42, 43]. This partly explains the political push and demand to rapidly transfer functions to counties in 2013 even before counties had appropriate structures to undertake these functions. Notably though, is that the health sector has traditionally not been involved in the design of decentralisation initiatives, but rather left to play “catch-up” through adapting to broader changes [24].

In analysing the capacity of the Kilifi CDoH, we find that during the early implementation of devolution the CDoH inherited the existing hardware elements including physical infrastructure for delivering health services and a good financial resource allocation from the County Treasury in 2013/14. However, during that year, the department’s tangible and intangible software elements were significantly lacking. There was no clear organisational structure with clear mandates and roles for different actors, and the existing managers lacked the knowledge and skills required for the strategic planning and budgeting roles. In addition, by the time of the 2013/14 fiscal year resource bidding process at the county level, the CDoH did not have a sector strategic plan and an AWP, both of which are essential tangible software elements required to influence and facilitate the resource bidding at county level. In terms of intangible software, we find that the CDoH lacked the necessary power, appropriate relationships and communication capacity to influence the decision by the county treasury to direct that hospital to close their individual user fee bank accounts and deposit all user fee funds into the county consolidated revenue account. Barasa et al. (2016) reported that hospital user fees are the single most important source of readily available financial resources for addressing many recurrent and often emergency needs within Kenyan county hospitals [44, 45]. The loss of this source of revenue coupled with the perverse re-centralisation of financial management processes meant that the hospitals could not address their emergency recurrent needs appropriately. Our findings support Omar’s (2002) assertion that health system decentralisation always brings additional responsibilities including of planning and resource allocation to local decision makers, yet their capacity for undertaking these responsibilities is often lacking, and often ignored during the design and implementation of decentralised systems [22].

From our findings we see that, during the county planning and budgeting process, the different county departments, including the CDoH are supposed to use their annual priorities as indicated in their AWPs to compete for the county revenue in a bidding process that is coordinated by the county treasury [46]. However, we see that during the 2013/14 fiscal year the Kilifi CDoH did not participate in the resource bidding process at the county level. This forced the county treasury to allocate the CDoH resources based on a generic budget. As highlighted above, the reasons for the CDoH non-participation in the resource bidding process were lack of significant tangible software capacity elements including lack of a clear organisational structure, failure to appoint key offices to drive the department’s budgeting process, and lack of a departmental strategic plan and AWP to guide the annual sector priorities. This lack of appropriate capacity reduced the CDoH’s ability to influence the amount of resources allocated to the health sector, thus reducing its decision space over resource allocation [4].

In line with the spirit of decentralisation within the 2010 constitution, county departments are mandated to further decentralise decision making particularly of planning, budgeting, and financial management related to day-to-day service delivery to sub-county management entities [39]. The lack of appropriate structure within the CDoH meant that the department could not further decentralise its coordination and operational financial management roles to sub-county units including the hospital and sub-county management units. Subsequently all the hospital and sub-county management teams had to send in all of their requests for any purchases and expenditures to the Chief Officer at the county headquarters, causing a significant delay in implementation of planned activities. This happened even though before the implementation of county governments, district and hospital management units had a delegated accounting mandate and managed their local budgets for operational recurrent expenditures [5, 25, 26]. It thus can be argued that the implementation of devolution reversed historical gains of health sector financial management processes at the peripheral units at least in the short term.

In general, our findings highlight the necessity for proper and appropriate structures and capacity at peripheral level in decentralised health systems, if they are to succeed in taking up the functions mandated to them. This finding present an essential contribution into the existing literature which has been largely silent in reporting on local level capacity while highlighting health sector decentralisation outcomes [7, 32].

In addition to enhancement of technical efficiency, one of the most common reasons for the promotion of public sector decentralisation is the belief that it enhances accountability largely through supporting community participation in decision making [10]. In analysing the accountability structures and practices at the county level, we see that the CA, composed of elected MCAs, is the main organ that offers financial and political accountability oversight of the broader county government [39]. The devolution laws also created and mechanism for direct public participation in the county level planning and budgeting process. From our results, we see that though there was an attempt to invite public views and comments when the overall county budget had been finalised, there was no public involvement at the health sector level as the health sector planning structures had not been established, hence the process did not happen as expected. In addition, our findings reveal lack of appropriate knowledge and skills among the MCAs meant that they we unable to appropriately carry out their accountability function. This highlights the important interactions between accountability mechanisms and organisational capacity, and agrees with the observations of Bossert and Mitchel (2011) [32].

The national government, due to political pressure was forced by the county governments to decentralise all county the functions immediately even though the law provided for up-to three years for the transfer of functions to be undertaken. This example highlights that, the national government had less software tangible elements of ‘power’ over the county governments in influencing the process of transfer of functions. Subsequently because of the counties exuded more political power, they were able to expand their decision space over the transfer of county functions process.

## Study strengthens and limitations

The embedded nature of this study, owing to BT’s ‘insider’ position both at the national MoH and Kilifi County levels, and the nesting of the study within the Kenyan learning site, facilitated access to individuals and in-depth learning, and enhanced the opportunity for the study findings to be regularly shared with relevant decision makers.

A key limitation was the collection of data during the early days of devolution implementation. This meant that many of the effects were still unfolding. The findings therefore only relate to the early health system experiences of devolution in Kenya. However, the changes observed during these early days of devolution point to important issues that may persist into the medium and possibly even longer term in the absence of further intervention. An example is the recentralisation of financial management control. In addition, the study findings are important because understanding the effects on and functioning of the health system during such radical change has important lessons for other countries planning devolution or other large scale change. It would be naïve to consider that health systems are ever static – in fact they are usually undergoing some form of change, albeit less dramatic than Kenyan devolution [47].

The primary focus on one county experiences could also be viewed as a limitation of the study. However, the decision to use one county was deliberate, as it allowed for a deeper exploration of the issues under focus within the study, by involving a broad range of stakeholders. Similar findings on the influences on the county level experiences in Kilifi, like the effect by rushed transfer of function, delays in setting up county level structures and low capacity of county level stakeholders in undertaking their mandates, have also been reported in other counties [48, 49]. In addition, data collection for this study was also done at a national level through interviews and observations, and this allowed for drawing on country-wide lessons in interpreting the findings from Kilifi county.

## Study conclusions and recommendations

The findings from this study show the relevance and value of our conceptual framework. The framework also offers highlights key issues that should be addressed when designing and implementing decentralization processes.

For Kenya, we recommend interventions to progressively improve county level capacity for health sector planning, budgeting and financial management, and the functioning of county level community involvement and accountability structures. We argue that if the county level capacity is improved, then the opportunities created by devolution for improving county level health sector planning and budgeting and community involvement will be harnessed. An example of capacity enhancing strategy could include integrating policy evaluation research during times of major health sector policy implementation. This would enhance the ability of providing real-time understanding of the policy implementation effects and provide a feed-back mechanism for enhancing policy implementation.

From this study for example, when we shared our preliminary findings with the different actors at county level, concerned actors came together to start deliberations of developing county level legislation, dubbed *The Kilifi County Facility Improvement Fund Bill*. This bill - which is currently before the county assembly - will allow the CDoH to collect and retain user fees in hospitals and utilise them for improving their health facilities. In addition, from our continued interaction with the Kilifi CDoH within the Kilifi learning site, there was an observed better participation of the CDoH in the planning and budgeting process in the 2014/15 fiscal year, which also had more community involvement from facility level. However, whether the final 2014/15 CDoH budget was more aligned with the sector specific priorities is yet to be established

The Kenyan experience reported here reminds policymakers in other settings of the importance of establishing, as integral to a decentralisation strategy, clear and distinct roles between the centre and the periphery. The experience also shows the importance of building the knowledge and skills among the different actors. Finally, it emphasises the need for health sector policy actors to be aware of the broader political context when designing and implementing technical strategies for health sector decentralisation.

## Additional file

Additional file 1: Table S1. Summary of key national level planning and budgeting events relevant to the health sector planning process. **Table S2.**Summary of the key county planning and budgeting events relevant to the CDoH planning process (DOCX 29 kb)
