# Supplementary material for: How does decentralisation affect health sector planning and financial management? a case study of early effects of devolution in Kilifi County, Kenya
Source: Int J Equity Health. 2017 Sep 15;16:151. doi: 10.1186/s12939-017-0649-0 (PMC5599897; doi:10.1186/s12939-017-0649-0)
Supplement: Additional file 1: Table S1. — Summary of key national level planning and budgeting events relevant to the health sector planning process. Table S2.Summary of the key county planning and budgeting events relevant to the CDoH planning process (DOCX 29 kb) [file 12939_2017_649_MOESM1_ESM.docx]

**Additional file 1**

***Table S1: Summary of key national level planning and budgeting events relevant to the health sector planning process***

| Key event | Time line | Responsible Person/Institution | Comments |
| --- | --- | --- | --- |
| Elaboration of newly elected government five year Medium Term Plan (MTP) | End of first September after the election | National Treasury | New government aligns its campaign manifesto with the Kenya Vision 2030 National long-term strategic development plan |
| Publishing of budget guidelines to all government entities | 30th August each year | Treasury Cabinet secretary | Guidelines outline respective year’s priorities and sets broad ceilings for government entities e.g. ceilings on allocations between recurrent and development expenses. Guidelines will be used by controller of budgets to appraise budgets from all government entities. |
| Submission of Budget Review and Outlook Paper (BROP) to cabinet for approval | 30th September each year | Treasury Cabinet secretary | Outlines government budgetary performance for previous financial year and presents government projected revenue for coming financial year, providing indicative allocations to all government sectors including county governments |
| Submission of annual budget policy statement to parliament for approval | 15th February each year | Treasury Cabinet secretary | Policy statement outlines broad national strategic priority goals which national and county government entities should align their budgets to. |
| Submission of government budget estimates to parliament budget committee | 30th April each year | Treasury Cabinet secretary | National assembly scrutinizes allocations to national government entities in-line with governed strategic priorities.  Senate scrutinizes allocations to county governments in-line with constitutional requirements and overall government strategic priorities |
| Parliament approves government budget estimates | 30th June each year | National Assembly | Sets stage for development of appropriation bill to allow government to draw funds from the consolidated fund to implement the budget. |

***Table S2: Summary of the key county planning and budgeting events relevant to the CDoH planning process***

| **Key event** | **Timeline** | **Responsible Person/Institution** | **Comments** |
| --- | --- | --- | --- |
| Development of county level sector specific Strategic Plans | End of first September after the election | Chief Officer of respective County Department | CoDH develops its five year Strategic plan, aligned to the Kenya Health Sector Strategic Plan |
| Consolidation of sector strategic plan to develop the Consolidated County Integrated Development Plan (CIDP) | End of first September after the election | County Treasury | County government aligns its CIDP with its campaign manifesto and with the National government MTP and Kenya Vision 2030 |
| County Budget Review and Outlook Paper submitted to county assembly for approval | 30th September each year | County Treasury | Outlines county government’s budgetary performance for previous financial year and presents projected revenue for coming financial year, providing indicative allocations to all sectors and departments in the county |
| Resource biding and allocation by respective county departments | October – December each year | All Departmental Chief Officers | CDoH having undertaken its review of previous year’s performance undertakes bidding/lobbying for an increase or maintenance of budgetary allocation |
| County fiscal strategy paper submitted to county assembly for approval | 15th February each year | County Treasury | Outlines broad county fiscal strategic priority goals of which county departments should align their budgets to |
| Submission for review of county budget estimates to County Assembly budget committee | 30th April each year | County Treasury | County assembly scrutinizes proposed allocations and expenditure to all entities. Invites public participation in this process |
| Approval of county budget | 30th June every year | County Assembly | Sets stage for development of county appropriation bill to allow county government to draw funds from the consolidated county revenue fund to implement the budget |
